# The Impact of Heterologous Regulatory Genes from Lipodepsipeptide Biosynthetic Gene Clusters on the Production of Teicoplanin and A40926

**DOI:** 10.3390/antibiotics13020115

**Published:** 2024-01-24

**Authors:** Kseniia Zhukrovska, Elisa Binda, Victor Fedorenko, Flavia Marinelli, Oleksandr Yushchuk

**Affiliations:** 1 Department of Genetics and Biotechnology, Ivan Franko National University of Lviv, 79005 Lviv, Ukraine; kseniya-oksana.zhukrovska@lnu.edu.ua (K.Z.); viktor.fedorenko@lnu.edu.ua (V.F.); oleksandr.yushchuk@uninsubria.it (O.Y.); 2 Department of Biotechnology and Life Sciences, University of Insubria, 21100 Varese, Italy; elisa.binda@uninsubria.it

**Keywords:** ramoplanin, chersinamycin, teicoplanin, A40926, production improvement, pathway-specific regulatory genes, antibacterial activity, soil microorganisms

## Abstract

StrR-like pathway-specific transcriptional regulators (PSRs) function as activators in the biosynthesis of various antibiotics, including glycopeptides (GPAs), aminoglycosides, aminocoumarins, and ramoplanin-like lipodepsipeptides (LDPs). In particular, the roles of StrR-like PSRs have been previously investigated in the biosynthesis of streptomycin, novobiocin, GPAs like balhimycin, teicoplanin, and A40926, as well as LDP enduracidin. In the current study, we focused on StrR-like PSRs from the ramoplanin biosynthetic gene cluster (BGC) in *Actinoplanes ramoplaninifer* ATCC 33076 (Ramo5) and the chersinamycin BGC in *Micromonospora chersina* DSM 44151 (Chers28). Through the analysis of the amino acid sequences of Ramo5 and Chers28, we discovered that these proteins are phylogenetically distant from other experimentally investigated StrR PSRs, although all StrR-like PSRs found in BGCs for different antibiotics share a conserved secondary structure. To investigate whether Ramo5 and Chers28, given their phylogenetic positions, might influence the biosynthesis of other antibiotic pathways governed by StrR-like PSRs, the corresponding genes (*ramo5* and *chers28*) were heterologously expressed in *Actinoplanes teichomyceticus* NRRL B-16726 and *Nonomuraea gerenzanensis* ATCC 39727, which produce the clinically-relevant GPAs teicoplanin and A40926, respectively. Recombinant strains of NRRL B-16726 and ATCC 39727 expressing *chers28* exhibited improved antibiotic production, although the expression of *ramo5* did not yield the same effect. These results demonstrate that some StrR-like PSRs can “cross-talk” between distant biosynthetic pathways and might be utilized as tools for the activation of silent BGCs regulated by StrR-like PSRs.

## 1. Introduction

Filamentous actinobacteria, also known as actinomycetes, represent one of the most abundant natural sources of antibiotics [1]. Soil-dwelling, Gram-positive actinobacteria possess large genomes (of up to 13 Mbp [2]) rich in GC content. These genomes harbor numerous biosynthetic gene clusters (BGCs), which consist of structural and regulatory genes governing the synthesis of antibiotics and other specialized metabolites [3]. It is noteworthy that, within a single genome, only a few BGCs are active, while others remain “silent” under typical laboratory conditions [4]. Various families of pathway-specific transcriptional regulators (PSRs), encoded by cluster-situated regulatory genes (CSRGs), play a crucial role in regulating the expression of antibiotic BGCs [5]. Among these regulators are *Streptomyces* antibiotic regulatory proteins (SARPs), which, as classic examples of PSRs, control the biosynthesis of diverse antibiotics [6]. SARPs have been extensively studied and leveraged as powerful tools to activate silent BGCs in *Streptomyces* spp. [7,8,9]. Another known group of PSRs involved in the regulation of antibiotic biosynthesis is the LuxR family transcriptional regulators [10]. LuxR family proteins were reported to control the biosynthesis of polyene antifungals, such as nystatin, natamycin, amphotericin, and others [11,12,13]. The third relevant group of PSRs, known as StrR-like transcriptional regulators, have received less attention in terms of systematic in silico analyses and structural studies than SARPs and LuxR family PSRs. The founding member of this group is the eponymous StrR, a PSR associated with the streptomycin and hydroxystreptomycin BGCs in *Streptomyces griseus* ssp. *griseus* NBRC 13350 and *Streptomyces glaucescens* GLA.0 [14,15]. Subsequently, another StrR-like PSR, NovG, was demonstrated to activate the production of the aminocoumarin antibiotic novobiocin [16]. StrR-like PSRs were then investigated as regulators of glycopeptide antibiotic (GPA) biosynthesis [17]. Initially, StrR was predicted to carry a helix-turn-helix (HTH) DNA-binding domain in the central region [15], but more recent annotations noted the presence of a ParB-like nuclease domain (characteristic of the Spo0J superfamily of chromosome segregation proteins [18]) at the N-terminus [19]. To date, further investigations are needed to better define the structure of StrR-like regulatory proteins, their mode of DNA-binding, the role of the ParB-like nuclease domain, as well as their phylogeny and evolution.

GPAs represent a clinically successful class of cell wall biosynthesis inhibitors (via binding the lipid II) produced by actinomycetes [20,21]. The first-generation GPAs, vancomycin and teicoplanin, are naturally produced by various strains of *Amycolatopsis orientalis* and *Actinoplanes teichomyceticus* NRRL B-16726, respectively [22,23]. In contrast, the semisynthetic second-generation GPAs telavancin, oritavancin, and dalbavancin are chemically modified derivatives of the natural GPAs vancomycin, chloroeremomycin (a vancomycin-like GPA produced by *Kibdelosporangium aridum* A82846 [24]), and A40926 (a teicoplanin-like GPA produced by *Nonomuraea gerenzanensis* ATCC 39727 [25]), respectively. Many other natural and semisynthetic GPAs are known but they are not clinically used [20,26]. Although every known GPA BGC carries a gene for an StrR-like PSR [27], the most studied are *bbr* (from the balhimycin BGC in *Amycolatopsis balhimycina* DSM 5908) [28,29,30], tei15* from the teicoplanin BGC (*tei*) [31,32,33], and *dbv4* from the A40926 BGC (*dbv*) [30,34,35]. All these genes appear to encode key activators of GPA biosynthesis, and their knocking out resulted in the complete abolishment of antibiotic production [32,35]. This feature has allowed the use of *bbr*, tei15*, and *dbv4* as powerful tools to engineer GPA-overproducing strains, either in their native or in heterologous hosts, or to activate silent biosynthetic pathways for GPAs [19,29,32,36].

GPAs appear to share a genetic relationship with another significant group of lipid II binders known as ramoplanin-like lipodepsipeptides (LDPs) [37]. LDPs include ramoplanin (produced by *Actinoplanes ramoplaninifer* ATCC 33076 [38,39]), enduracidin (produced by *Streptomyces fungicidicus* B 4477 [40,41]), and the recently described chersinamycin (produced in *Micromonospora chersina* DSM 44151 [42]). Among the three, the most advanced in clinical trials is ramoplanin due to its potent bactericidal activity against aerobic and anaerobic Gram-positive bacteria, including methicillin-resistant enterococci (MRE), vancomycin-resistant enterococci (VRE), and *Clostridiodes difficile* [43,44]. Ramoplanin has progressed to phase III clinical trials for the specific treatment of gastrointestinal infections caused by VRE and *C. difficile* strains [45,46,47]. GPA and LDP BGCs share similar genes, as those involved in the biosynthesis of nonproteinogenic amino acids 3,5-dihydroxyphenylglycine and 4-hydroxyphenylglycine [37], exporter genes [46], and CSRGs coding for StrR-like PSRs. These genes, identified in the ramoplanin (*ramo*), chersinamycin (*chers*), and enduracidin (*end*) BGCs, are named *ramo5* [47], *chers28* [42], and *end22* [41], respectively. The roles of *ramo5* and *chers28* have not been experimentally investigated yet, while *end22* was demonstrated to activate enduracidin production [48]. Indeed, the knockout of *end22* abolished enduracidin production, while its overexpression led to a several-fold increase in enduracidin titers [48].

This motivated our interest to investigate in this study the phylogenetic position of LDP StrR-like PSRs and their structural features in silico, assessing their relationships with better-studied regulators of GPA biosynthesis. Furthermore, we studied the properties of *ramo5* from *A. ramoplaninifer* ATCC 33076 and its ortholog *chers28* from *M. chersina* DSM 44151, heterologously expressing them in the producers of the clinically valuable GPAs teicoplanin and A40926 (*A. teichomyceticus* NRRL B-16726 and A40926 *N. gerenzanensis* ATCC 39727, respectively). After investigating the GPA production dynamics in the recombinant strains, we demonstrated that *chers28* enhances teicoplanin and A40926 production under specific expression conditions. Our results indicate that StrR-like PSRs of the GPA and LDP pathways are conserved enough to “cross-talk”, expanding the genetic toolkit for creating GPA-overproducing strains.

## 2. Results

### 2.1. Phylogenetic Relationships between StrR-like Regulators Coded within Antibiotic BGCs

Considering the presence of similar genes for StrR-like PSRs in LDP and GPA BGCs, we decided to investigate the phylogenetic relationships between these PSRs and collocate them in the broader context of the phylogeny of StrR-like PSRs coded within available antibiotic BGCs. To establish a set of proteins for phylogenetic reconstruction, we initially analyzed eight publicly available LDP BGCs [39,41,42] in search of Ramo5 homologs (Table S1A). Surprisingly, we found that *end* from *S. fungicidicus* ATCC 21013 as well as BGCs for putative LDPs from *Streptomyces* sp. SLBN-134, *Am. orientalis* DSM 40040 and B-37, and *Am. balhimycina* DSM 44591 carried two genes for StrR-like PSRs (Table S1A). Although *end22* (ABD65942) was experimentally investigated [48], the gene for the second StrR-like PSR—*end24* (ABD65944)*—*was probably missed before.

In the next step, we analyzed all the antibiotic BGCs from *Actinomycetota* available in the MIBiG database [49] at the time of the manuscript preparation (November 2023), searching for CSRGs for StrR-like PSRs (Table S1B). In addition to the 12 StrR-like PSRs found in LDP BGCs and Ramo5, we identified 52 StrR-like PSRs encoded in BGCs for different types of natural compounds including GPAs, aminoglycosides, polyketides, aminocoumarins, terpenes, etc. (Table S1B). Finally, we added to our phylogenetic reconstruction sequences of 19 StrR-like PSRs found in some well-known GPA BGCs, which were absent in MIBiG (Table S2). In the end, our set of sequences consisted of 83 StrR-like proteins (Tables S1A,B and S2).

**Figure 1:**
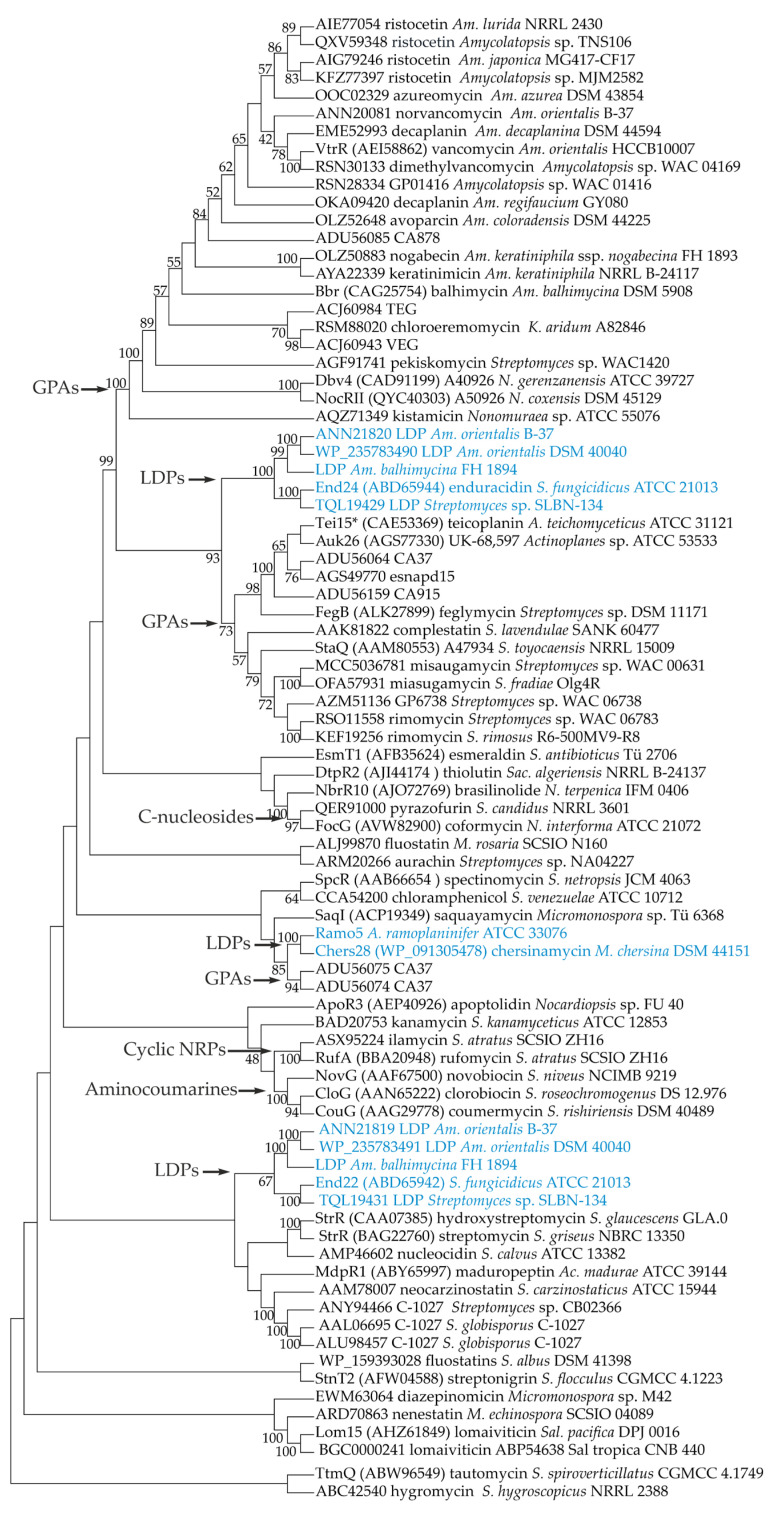
Unrooted maximum-likelihood phylogenetic tree of 83 StrR-like PSRs coded within GPA, LDP (highlighted in blue), and other antibiotic BGCs. Branch lengths were ignored (the tree is represented as a cladogram), and numbers at the nodes show bootstrap support (inferred from 500 bootstraps) of the clade (only those of >40% are shown). Phylogenetic reconstruction was performed using Mega 11 (v. 11.0.13) [50].

The maximum likelihood phylogeny of StrR-like PSRs appeared to be quite puzzling (Figure 1). StrR-like PSRs of LDP BGCs formed three distinct clades within the tree. The first clade was formed by End24 orthologs and resided inside StrR-like PSRs coded within GPA BGCs (Figure 1). End22 orthologs were located in a separate clade (Figure 1). This evidence indicates that *end22* and *end24* did not coexist within the *end* BGC as a result of a gene duplication event, but they were likely acquired independently. The same conclusion seems true for the other pairs of StrR-like regulators coded within the putative LDP BGCs from *Am. orientalis* B-37 and DSM 40040, *Am. balhimycina* DSM 44591, and *Streptomyces* sp. SLBN-134.

Ramo5 and Chers28 formed yet another clade, grouping together with the two StrR-like PSRs coded within the putative GPA BGC from the environmental isolate CA37 (Figure 1). The Ramo5–Chers28–CA37 clade indeed appeared to be distant from the clade grouping most of the GPA StrR-like PSRs.

Additional well-supported clades of the tree were observed for StrR-like PSRs of nenestatin and lomaiviticin BGCs, enedyine BGCs, aminocoumarin BGCs, ilamycin and rufomycin BGCs, and C-nucleoside BGCs (Figure 1).

We further decided to compare the amino acid (aa) sequences of the StrR-like PSRs coded in LDP BGCs with the founding member of StrR-like regulators—the StrR from *S. griseus* ssp. *griseus* NBRC 13350. StrR was initially described as a DNA-binding protein, carrying an HTH DNA-binding domain (in the middle section of the protein) [15]. Further experiments indicated a non-identified C-terminal domain, which is dispensable for DNA-binding in vitro but important for in vivo functioning [51]. We used in silico tools for the prediction of conserved domains, as well as the secondary and 3D structures of StrR, to characterize it better and use it as a prototype for the analysis of StrR-like regulators coded in GPA and LDP BGCs.

**Figure 2:**
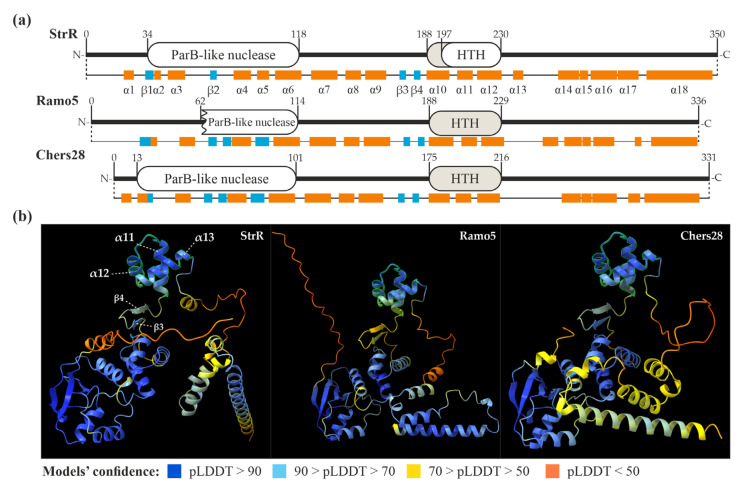
Domain architectures and secondary structures of StrR, Ramo5, and Chers28 (**a**) as well as models of their tertiary structures (tri-helical HTH DNA-binding domains are highlighted in (**b**), as predicted with CD-Search [52] and AlphaFold (visualized using Chimera X) [53,55].

First, we analyzed the amino acid sequence of StrR using the conserved domain identification tool CD-Search [52]. This yielded specific hits for two domains: a ParB-like nuclease domain (34–118 aa) and an AsnC-like HTH DNA-binding domain (197–230 aa) (Figure 2a). The latter overlapped with the position (207–227) of an HTH motif initially identified in StrR [51]. Subsequently, we predicted the secondary and 3D structures of StrR using AlphaFold v2 [53] with Chimera X (v. 1.4) [54,55]. Within StrR, 18 α-helices and 4 β-strands were identified (Figure 2a). Notably, α10-12 corresponded to the HTH DNA-binding domain as identified with CD-Search. The model of the 3D structure of StrR (Figure 2a) revealed that α10-12 (189–230 aa, predicted with very high confidence) form a DNA-binding domain, which could be classified as a simple tri-helical HTH (Figure 2b) [56]. The 3D structure also indicated an N-terminal ParB-like nuclease domain, which was modeled with high confidence (Figure 2b). The putative C-terminal domain, believed to be involved in RNA-polymerase recruitment [51], appeared to be a combination of several α-helices, which were predicted with low confidence (Figure 2b).

We applied the same approach to predict the secondary and 3D structures of Ramo5 and Chers28. CD-Search, however, was unable to predict the DNA-binding domains in these proteins, although the secondary and tertiary structure predictions yielded the same tri-helical HTH DNA-binding domains as in StrR (Figure 2a,b). Overall, the predicted secondary and tertiary structures of Ramo5 and Chers28 were similar to those of StrR (Figure 2), but in Ramo5, the ParB-like nuclease domain was predicted as being truncated with CD-Search (Figure 2a). Finally, we compared the secondary and tertiary structures of StrR, Ramo5, and Chers28 with those of Bbr, Dbv4, Tei15*, FegB, StaQ, FocG, RufA, NovG, AAL06695, and Lom15 (representing other clades of the tree, as shown in Figure 1 and Figure S1). Strikingly, we found out that FocG (AVW82900), coded in the coformycin BGC of *N. interforma* ATCC 21072 (Table S1B), completely lacked a DNA-binding domain (Figure S1). As FocG belonged to the clade grouping StrR-like PSRs from C-nucleoside BGCs (Figure 1), we analyzed the structures of its counterparts, NbrR10 and QER91000 (coded in the brasilinolide BGC from *N. terpenica* IFM 0406 and the pyrazofurin BGC from *S. candidus* NRRL 3601, respectively, as shown in Table S1B), as well as the clades’ outgroup, DtpR2 (coded in the thiolutin BGC of *Sac. algeriensis* NRRL B-24137, as shown in Table S1A) (Figure 1). The HTH DNA-binding domain appeared to be lost in all members of the “C-nucleoside” clade, but it was present in the outgroup (Figure S1). Apart from FocG, NbrR10, and QER91000, all the other analyzed sequences demonstrated similar secondary and tertiary structures, including the conserved architecture of the N-terminal ParB-like nuclease domain. As another conserved feature, two short antiparallel β-strands were found at the N-terminal side of the HTH DNA-binding domain (corresponding to the β3 and β4 of StrR) (Figure S2). The putative C-terminal domains of all these proteins consisted of α-helices predicted with low confidence but shared aa sequence identity (Figure S2).

### 2.2. Expression of Cluster-Situated Regulatory Genes ramo5 and chers28 in N. gerenzanensis ATCC 39727

**Figure 3:**
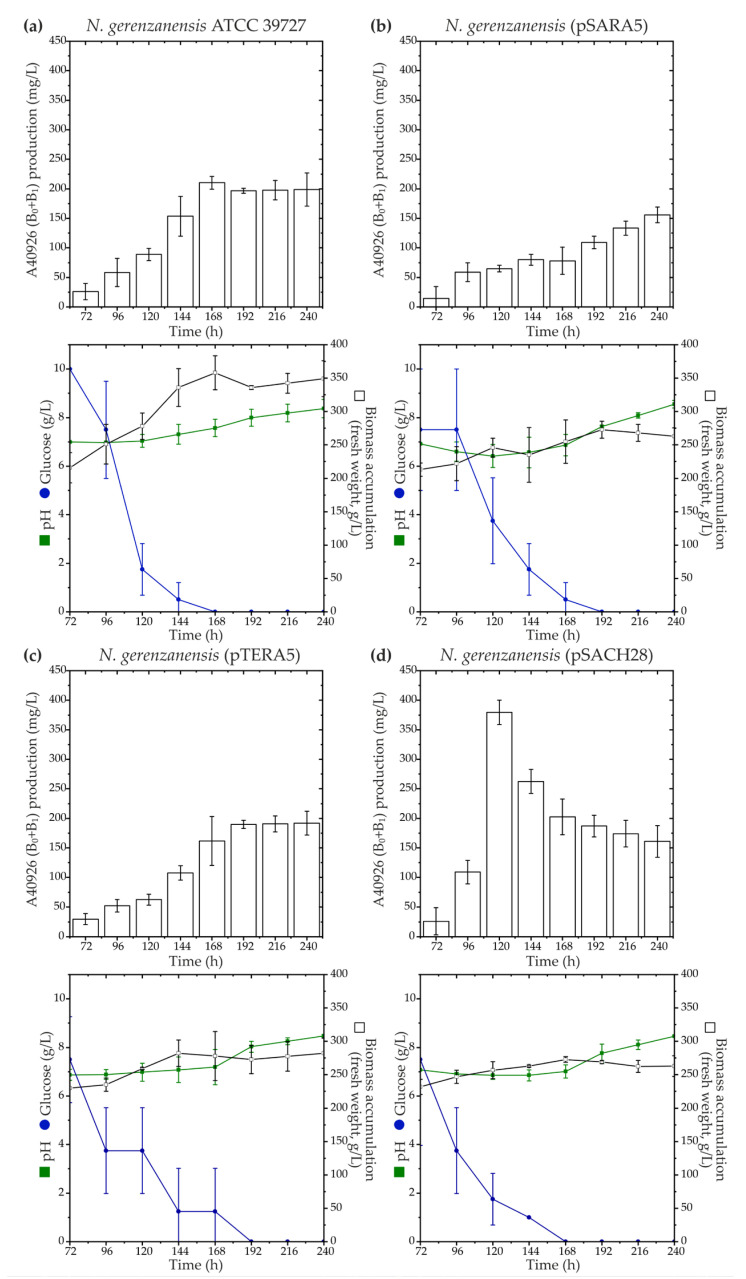
Production time courses of A40926 (factors B_0_ + B_1_) along with key growth parameters such as biomass accumulation, pH, and glucose consumption for *N. gerenzanensis* ATCC 39727 (**a**), as well as recombinant strains expressing *ramo5* and *chers28*: *N. gerenzanensis* (pSARA5) (**b**), (pTERA5) (**c**), and (pSACH28) (**d**). Comparison with ATCC 39727 wild type is shown since *N. gerenzanensis* (pSET152A) and (pTES) had the same production and cultivation parameters as the parental strain. The results are presented as mean values from three independent experiments with error bars representing ±2SD.

According to our phylogenetic reconstruction, Ramo5 and Chers28 belonged to a clade different from the native StrR-like PSRs coded in *dbv* and *tei*, although the corresponding LDP and GPA BGCs were previously found to be related [37]. To investigate whether StrR-like PSRs involved in the regulation of LDP biosynthesis could impact GPA biosynthetic pathways, we expressed *ramo5* and *chers28* in *N. gerenzanensis* ATCC 39727 using two platforms—pSET152A [33] and pTES plasmids [57]—where the expression was driven by *aac(3)IV* and *ermE* promoters, respectively. Both promoters were previously shown to function in *N. gerenzanensis*, with *aac(3)IVp* proving to be significantly more active in driving gene expression [36]. Recombinant vectors, namely, pSARA5, pTERA5, pSACH28, and pTECH28 (see Section 4), were transferred to *N. gerenzanensis* ATCC 39727, generating *N. gerenzanensis* (pSARA5), (pTERA5), (pSACH28), and (pTECH28). Subsequently, we assessed A40926 production using HPLC in all the recombinant strains grown in the industrial production medium FM2, as previously described [58] (Figure 3).

The expression of *ramo5* did not yield a positive effect on A40926 production in the recombinant strains (Figure 3a–c). We observed that *N. gerenzanensis* (pSARA5) and (pTERA5) accumulated less biomass than the parental strain (*N. gerenzanensis* ATCC 39727). While *N. gerenzanensis* (pTERA5) produced A40926 at the level of the parental strain, (pSARA5) seemed to display reduced A40926 production (Figure 3b). Conversely, the expression of *chers28* under the control of *aac(3)IVp* demonstrated a positive impact on A40926 production. Despite *N. gerenzanensis* (pSACH28) accumulating less biomass than the parental strain, it exhibited a peak production of ca. 400 mg/L of A40926 after 120 h of cultivation (Figure 3d). In comparison, the parental strain produced ca. 100 mg/L of the antibiotic after the same cultivation time, reaching a production peak of ca. 200 mg/L after 168 h of growing (Figure 3a). Unexpectedly, *N. gerenzanensis* (pTECH28) showed very scarce growth, wherein the pH of the cultural suspension dropped to pH 5.0, and the strain did not produce any detectable amounts of the antibiotic.

### 2.3. Expression of ramo5 and chers28 in A. teichomyceticus NRRL B-16726

**Figure 4:**
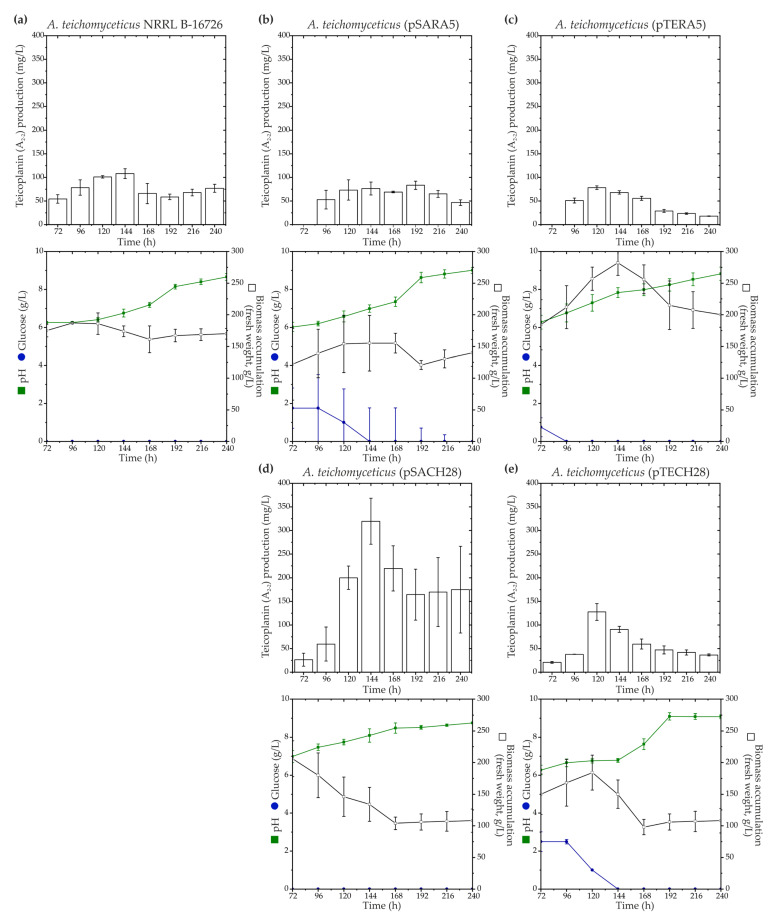
Time courses of teicoplanin (factor A_2-2_) production along with key growth parameters such as biomass accumulation, pH, and glucose consumption for *A. teichomyceticus* NRRL B-16726 (**a**), as well as recombinant strains expressing *ramo5* and *chers28*: *A. teichomyceticus* (pSARA5) (**b**), (pTERA5) (**c**), (pSACH28) (**d**), and (pTECH) (**e**). Comparison with NRRL B-16726 wild type is shown since *A. teichomyceticus* (pSET152A) and (pTES) had the same production and cultivation parameters as the parental strain. The results are presented as mean values from three independent experiments with error bars representing ±2SD.

To investigate the influence of *ramo5* and *chers28* on teicoplanin biosynthesis, aforementioned recombinant plasmids, namely, pSARA5, pTERA5, pSACH28, and pTECH28, were introduced into *A. teichomyceticus* NRRL B-16726. Both *aac(3)IVp* and *ermEp* were previously employed for robust gene expression in *A. teichomyceticus* NRRL B-16726, with *aac(3)IVp* being the best-performing one [33]. All the recombinant strains were cultivated in parallel with the parental *A. teichomyceticus* NRRL B-16726 in the industrial production medium TM1 previously described in [59], and teicoplanin production levels were assessed (Figure 4).

Similar to what was observed in *N. gerenzanensis* ATCC 39727, the expression of *ramo5* did not positively impact teicoplanin production. *A. teichomyceticus* (pSARA5) exhibited reduced biomass accumulation (Figure 4a,b), whereas *A. teichomyceticus* (pTERA5) showed increased biomass accumulation compared with the parental strain (Figure 4a,c).

In both the recombinants, teicoplanin production remained at the level of the parental strain (Figure 4a–c). On the other hand, as in *N. gerenzanensis* ATCC 39727, the expression of *chers28* had a positive effect on teicoplanin production, albeit dependent on the used expression platform. *A. teichomyceticus* (pSACH28) and (pTECH28) both demonstrated reduced biomass accumulation compared with the parental strain (Figure 4d,e), but only *A. teichomyceticus* (pSACH28) produced a higher amount of teicoplanin. *A. teichomyceticus* (pSACH28) achieved a peak in teicoplanin production after 144 h of cultivation, yielding almost 400 mg/L of the antibiotic, whereas the parental strain produced ca. 110 mg/L of teicoplanin after the same cultivation time (Figure 4d).

## 3. Discussion

StrR-like PSRs were experimentally demonstrated to play a crucial role in activating gene expression in streptomycin BGCs [15], aminocoumarin BGCs [16,60], as well as GPA BGCs [28,32,35]. Our results indicate that StrR-like PSRs are broadly encoded within BGCs for many other classes of antibiotics, where they likely control their biosynthesis (Table S1). Comparative amino acid sequence analysis supported the assumption that all these transcriptional regulators are conserved and share a number of structural features (Figure S2). These features include a conserved N-terminal domain resembling the ParB-like nuclease domain, a tri-helical HTH DNA-binding domain, and a putative C-terminal domain composed of several α-helices (Figure S2). However, certain StrR-like proteins, including FocG, NbrR10, and QER91000 found within C-nucleoside antibiotic BGCs, seem to have lost their HTH DNA-binding domain. As a result, they are likely incapable of binding to DNA. Therefore, conducting experimental investigations on these proteins is important to ascertain whether they play any functional role in the biosynthesis of their respective antibiotics. Although the roles of both N-terminal and C-terminal domains in the function of StrR-like proteins remain poorly investigated, both seem to be important for proper protein functioning in vivo: the N-terminal domain might be involved in dimerization [19], while the C-terminal domain may play a role in RNA-polymerase recruitment [51].

Even if the phylogeny of StrR-like PSRs (Figure 1) lacked resolution (likely due to the rather small sampling), it nevertheless showed that StrR-like PSRs of LDP BGCs appear to be polyphyletic. Additionally, the well-studied enduracidin BGC, end [41], was found to carry an additional gene for StrR-like PSR, *end24*. While the known End22 serves as the activator of enduracidin biosynthesis [48], the role of the additional End24 in enduracidin biosynthesis remains unclear. The putative LDP BGCs of *Am. balhimycina* DSM 44591, *Am. orientalis* B-37, and DSM 40040 [42] also carry two genes for StrR-like PSRs (the *end22* and *end24* orthologs). Given that these strains are known producers of GPAs [42], and that the corresponding GPA BGCs also carry genes for StrR-like PSRs, this creates a paradox of three related StrR-like BGCs coexisting within a single strain. Thus, the distribution and phylogeny of StrR-like PSRs merit further investigation, especially considering that corresponding genes might serve as probes in the search for BGCs of novel compounds. Such analysis is currently underway in our laboratories.

As StrR-like regulators of ramoplanin and chersinamycin BGCs formed a separate clade on the phylogenetic tree (Figure 1), not closely related to other LDP or known GPA StrR-like PSRs, we focused on Ramo5 and Chers28 to investigate whether the heterologous expression of the corresponding genes might influence the production of the clinically relevant GPAs teicoplanin and A40926. Our results demonstrate that the heterologous expression of *ramo5* had no significant impact on either teicoplanin or A40926 production, while *chers28* improved the production of both these antibiotics when cloned under the *aac(3)IV* promoter. Notably, the impact of *chers28* overexpression varied depending on the expression platform used: the application of *aac(3)IVp* (in pSACH28) to drive gene expression resulted in an increased production of teicoplanin and A40926, while using *ermEp* (in pTECH28) did not give the same effect. These findings align with earlier research on promoter activity strengths in *A. teichomyceticus* NRRL B-16726 and *N. gerenzanensis* ATCC 39727 [33,36]. Furthermore, *chers28* expression positively influenced A40926 production, comparable to the expression of the native A40926 CSRG, *dbv4* [36]. Conversely, *chers28* expression had a less marked impact on teicoplanin biosynthesis compared with the expression of the native CSRG, tei15* [32].

The different effect of *chers28* and *ramo5* expression on GPA production is surprising since Ramo5 and Chers28 share 70% of the aa sequence identity. Notably, the “recognition” helices (the C-terminal helices of the HTH DNA-binding domains), responsible for precise protein–DNA interactions, were found to be identical in Ramo5 and Chers28 (Figure S2). However, other parts of the HTH DNA-binding domains, as well as the overall C- and N-terminal domains of Ramo5 and Chers28, were shown to be more divergent. The distinct properties observed in Ramo5 and Chers28 might also be attributed to the structural differences and amino acid sequence variations in their ParB-nuclease-like domains. Therefore, it cannot be excluded that any of these differences lead to more promiscuous DNA-binding properties in Chers28 than in Ramo5. However, further experimental evidence is necessary to clearly define the roles of the various domains within StrR-like PSRs. Whatever the real cause of the different properties of Ramo5 and Chers28 is, our results demonstrate that at least some StrR-like PSRs are conserved enough to activate antibiotic biosynthetic pathways governed by their distant homologs. The putative promiscuity of Chers28 suggests to consider this StrR-like PSR as a potential tool for the high-throughput activation of silent BGCs, following some available pioneering steps in this direction [7].

## 4. Materials and Methods

### 4.1. Bacterial Strains and Cultivation Conditions

All strains and plasmids utilized in this study are listed in Table 1. Genomic DNA extraction from *A. ramoplaninifer* ATCC 33076 and *M. chersina* DSM 44151 involved cultivation in Erlenmeyer flasks with 10 glass beads (∅5 mm) and 50 mL of ISP2 [61] liquid medium [61] for 72 h. *A. teichomyceticus* NRRL B-16726, *N. gerenzanensis* ATCC 39727, and their recombinant strains were routinely cultivated on ISP3 agar medium [61], supplemented with 50 µg/mL of apramycin sulfate when necessary, and incubated at 30 °C. Before DNA extraction, recombinant strains of *A. teichomyceticus* NRRL B-16726 and *N. gerenzanensis* ATCC 39727 were cultivated in 50 mL of ISP2 [61] liquid medium for 72 h.

For antibiotic production, all strains were cultivated in baffled Erlenmeyer flasks in an orbital shaker at 30 °C and 220 rpm. For teicoplanin production, *A. teichomyceticus* NRRL B-16726 and recombinant strains were initially cultivated in 50 mL of E25 preculture vegetative medium [59] for 72 h. Subsequently, 10% (*v*/*v*) of the preculture was inoculated into 100 mL of TM1 teicoplanin production medium [59]. *N. gerenzanensis* ATCC 39727 and its recombinant strains were cultivated in 50 mL of E26 vegetative medium [58] for 72 h. Then, 10% (*v*/*v*) of the preculture was inoculated into 100 mL of FM2 A40926 production medium [58]. Culture samples were collected at regular cultivation time intervals and analyzed to estimate biomass accumulation (fresh weight), glucose consumption (glucose concentration in culture broths was measured using Diastix sticks; Bayer AG, Leverkusen, Germany), and teicoplanin and A40926 production.

*E. coli* DH5α served as a general cloning host, while *E. coli* ET12567 (pUZ8002) [62] was used as a donor for conjugations with actinomycetes. *E. coli* strains were cultivated in lysogeny broth (LB) or in lysogeny broth agar (LA) containing 100 mg/mL of apramycin sulfate, 50 mg/mL of kanamycin sulfate, and 25 mg/mL of chloramphenicol when required. Antibiotics were obtained from Sigma-Aldrich (Merck Group, Darmstadt, Germany).

### 4.2. Extraction of the Genomic DNA

Genomic DNA extraction from *A. ramoplaninifer* ATCC 33076 and *M. chersina* NRRL B-24756 was carried out using the commercial NucleoSpin^®^ Microbial DNA Kit (Macherey-Nagel). For genomic DNA extraction from the recombinant strains of *A. teichomyceticus* NRRL B-16726 and *N. gerenzanensis* ATCC 39727, the salting-out procedure described in [63] was followed with some modifications. Mycelium was collected in 2 mL tubes and centrifuged for 1 min at 15,000× *g*. The supernatant was discarded, and the remaining wet biomass was resuspended in 450 µL of buffer (containing 25 mM of EDTA (pH 8.0) and 25 mM of Tris-HCl (pH 7.5), added with 4 mg of lysozyme) and incubated for 30 min at 37 °C. After incubation, 50 µL of 5M NaCl and 120 µL of SDS (10% (*w*/*v*)) were added, and the suspension was vigorously vortexed. The mixture was then incubated for 40 min at 65 °C and cooled to room temperature. Next, 240 µL of 5M CH_3_COOK was added, and the mixture was incubated at −20 °C for 15 min and centrifuged for 15 min at 15,000× *g*. The supernatant was transferred to a new 2 mL tube containing 750 µL of 100% (*v*/*v*) isopropanol. The mixture was vigorously vortexed, and the DNA was spooled onto a sealed Pasteur pipette and transferred to a new 1.5 mL tube containing 500 µL of 70% (*v*/*v*) ethanol. The DNA was rinsed, dried at 37 °C, and dissolved in sterile deionized water.

### 4.3. Generation of the Recombinant Plasmids

All plasmids used in this study are listed in Table 1. For the generation of recombinant plasmids, DNA fragments containing ORFs of *ramo5* and *chers28* were amplified from genomic DNA of *A. ramoplaninifer* ATCC 33076 and *M*. *chersina* NRRL B-24756, respectively, using Q5 High-Fidelity DNA Polymerase (NEB, Ipswich, MA, USA). The ramo5_F/R primer pair (Table 2) was employed for the amplification of *ramo5*, while *chers28* was amplified using the Mche_strR_F/R primer pair (Table 2).

To generate pSARA5, the 1054 bp fragment carrying the *ramo5* ORF was digested with the *Eco*RV restriction endonuclease and ligated with pSET152A, which was also digested with the same restriction endonuclease. Among the obtained recombinant plasmids, the one in which *ramo5* had the same direction as *aac(3)IVp* was selected using endonuclease restriction mapping. To generate pSACH28, the 1030 bp fragment carrying *chers28* ORF was digested with *Eco*RI/*Eco*RV restriction endonucleases and cloned via the same sites into the pSET152A, resulting in pSACH28. The plasmids pTERA5 and pTECH28 were generated in the same fashion, using the pTES plasmid as a base backbone. The obtained plasmids were verified with restriction mapping and sequencing.

### 4.4. Conjugal Transfer of Plasmids and Strain Verification

All plasmids listed in Table 1 were conjugally transferred to either *N. gerenzanensis* ATCC 39727 or *A. teichomyceticus* NRRL B-16726 using a previously described procedure [35,64,65]. Each constructed plasmid was individually transferred into the non-methylating *E. coli* ET12567 (pUZ8002) strain. The resulting derivatives were used as donor strains for intergeneric conjugation, with spores of *A. teichomyceticus* NRRL B-16726 or the mycelium of *N. gerenzanensis* ATCC 39727 serving as acceptors.

To obtain spore suspensions, *A. teichomyceticus* NRRL B-16726 lawns were grown on ISP3 agar for up to 168 h at 30 °C. Each lawn was flooded with deionized water, and the sporangia were scraped off the surface with a spatula. The collected mixture was vortexed and incubated in an orbital shaker at 30 °C for 1 h until the spores were released from the sporangia. Then, the obtained suspension was filtered through sterile cotton wool to remove vegetative mycelial fragments. Spores were concentrated by centrifugation (3220× *g* for 15 min) and resuspended in 1 mL of 15% (*v*/*v*) glycerol and stored at −80 °C. Approximately 10^6^ spores were mixed with 10^7^ of an overnight culture of donor cells and plated on ISP3 agar supplemented with MgCl_2_ (20 mM). After 12–16 h of incubation at 30 °C, each plate was overlaid with 1 mL of sterile deionized water containing 1.25 mg of apramycin sulfate and 750 µg of nalidixic acid. Transconjugants were selected as resistant to 50 µg/mL of apramycin sulfate.

To generate a fresh vegetative mycelium of *N. gerenzanensis* ATCC 39727 for conjugal transfer, one vial of WCB was inoculated into 50 mL of VSP medium (in a 250 mL Erlenmeyer flask with 10 glass beads of ∅5 mm) and cultured for 48 h in the orbital shaker at 30 °C and 220 rpm. After collecting and centrifuging (10 min, 3220× *g*) the mycelium, it was washed twice with sterile 20% (*v*/*v*) glycerol, resuspended in the same solution to a final volume of 20 mL, and kept at −80 °C. Then, 1 mL of the prepared mycelium suspension was mixed with approximately 10^9^ of an overnight culture of donor cells. The mixtures were plated on VM0.1 agar plates supplemented with 20 mM MgCl_2_. The plate overlaying and selection of transconjugants were performed as described previously for *A. teichomyceticus* NRRL B-16726.

Plasmid integration was verified by PCR using pAm_seq_F/R and pTES_ver_F/R primer pairs for pSET152A- and pTES-based plasmids, respectively.

### 4.5. Analysis and Quantification of A40926 and Teicoplanin Production

The extractions of teicoplanin and A40926 were performed as previously described in [58,59]. The antibiotics were extracted by mixing 1 volume of broth with 1 volume of borate buffer at pH 12.0. The samples from *A. teichomyceticus* strains fermentation were shaken on a rotary shaker at 200 rpm and 37 °C for 45 min, centrifuged for 15 min at 15,000× *g*, and the supernatants were used for quantitative analysis. The samples from *N. gerenzanensis* strains fermentation were centrifuged for 15 min at 15,000× *g*, and the supernatants were incubated for 1 h at 50 °C before analysis.

Quantitative analysis was performed by using the VWR Hitachi diode array L-2455 HPLC system, adhering to the methodology outlined in [65]. For teicoplanin, the detection was set at 236 nm, and for A40926, at 254 nm. 50 µL of each sample were injected into a 5 µm particle size Hypersil GOLD (Thermo Fisher Scientific, Waltham, MA, USA) column (4.6 by 250 mm). A40926 and teicoplanin were separated with a flow rate of 1 mL/min and a linear gradient of mobile phase B increasing from 15% to 64% over 30 min. Phase A was 32 mM HCOONH_4_ (pH 7.0)—CH_3_CN (90:10 (*v*/*v*))—and phase B was 32 mM HCOONH_4_ (pH 7.0)—CH_3_CN (30:70 (*v*/*v*)). 50 µL of teicoplanin (100 µg/mL) and A40926 (200 µg/mL) solutions from Sigma-Aldrich (Sigma-Aldrich, St. Louis, MO, USA) were used as standards. Since both A40926 and teicoplanin are produced as a complex of slightly different congeners [59,66], for A40926, the two main peaks (factors B_0_ + B_1_) were considered, whereas for teicoplanin, only the main peak (factor A_2-2_) was measured as previously reported [65].

### 4.6. Tools for In Silico Analysis

The MIBiG database was used as a source of antibiotic BGCs for the search for StrR-like PSRs [49]. Geneious 4.8.5 was employed for the routine analysis of amino acid and nucleic acid sequences [67]. Clustal Omega was used for multiple aa sequence alignments [68]. Phylogeny reconstruction was carried out using Mega11 (v.11.0.13) [69]. CD-Search was employed to detect conserved domain regions [70]. Chimera X (v.1.6.1) was used for AlphaFold-based prediction and visualization of the secondary and 3D structures of proteins [53,55,71].

## Figures and Tables

**Table 1:** Bacterial strains used in this work.

Plasmid	Description	Source or Reference
pSET152A	φC31-based integrative plasmid, pSET152 derivative carrying *aac(3)IVp* from pIJ773, Am^R^	[33]
pTES	φC31-based integrative plasmid, pSET152 derivative carrying *ermEp* flanked by *tfd* terminator sequences, Am^R^	[57]
pSARA5	pSET152A derivative carrying *ramo5* from ramoplanin BGC under the control of *aac(3)IVp*, Am^R^	This work
pSACH28	pSET152A derivative carrying *chers28* from chersinamycin BGC under the control of *aac(3)IVp*, Am^R^	This work
pTERA5	pTES derivative carrying *ramo5* under the control of *ermEp*, Am^R^	This work
pTEHC28	pTES derivative carrying *chers28* under the control of *ermEp*, Am^R^	This work
**Bacterial Strain**		
*A. ramoplaninifer*	Wild type, ramoplanin producer	ATCC 33076
*M. chersina*	Wild type, dynemicin and chersinamycin producer	NRRL B-24756
*A. teichomyceticus*	Wild type, teicoplanin producer	NRRL B-16726
*A. teichomyceticus* (pSET152A)	Wild type derivative carrying pSET152A	[32]
*A. teichomyceticus* (pSARA5)	Wild type derivative carrying pSARA5	This work
*A. teichomyceticus* (pSACH28)	Wild type derivative carrying pSACH28	This work
*A. teichomyceticus* (pTES)	Wild type derivative carrying pTES	This work
*A. teichomyceticus* (pTERA5)	Wild type derivative carrying pTERA5	This work
*A. teichomyceticus* (pTECH28)	Wild type derivative carrying pTECH28	This work
*N. gerenzanensis* ATCC 39727	Wild type, A40926 producer	ATCC 39727
*N. gerenzanensis* (pSET152A)	Wild type derivative carrying pSET152A	[36]
*N. gerenzanensis* (pSARA5)	Wild type derivative carrying pSARA5	This work
*N. gerenzanensis* (pSACH28)	Wild type derivative carrying pSACH28	This work
*N. gerenzanensis* (pTES)	Wild type derivative carrying pTES	This work
*N. gerenzanensis* (pTERA5)	Wild type derivative carrying pTERA5	This work
*N. gerenzanensis* (pTECH28)	Wild type derivative carrying pTECH28	This work
*E. coli* DH5α	General cloning host	MBI Fermentas, USA
*E. coli* ET12567 (pUZ8002)	(*dam-13::Tn9 dcm-6*), pUZ8002 (Δ*oriT*), used for conjugative transfer of DNA into actinomycetes	[62]

**Table 2:** Oligonucleotide used in this work. Restriction endonuclease recognition sites are underlined.

Name	Nucleotide Sequence (5′-3′)	Purpose
ramo5_F ramo5_R	TTTGATATCGGAGGGTTGGTATGGAGTCATTGCACATCG TTTGATATCGCCGCATTCGCTGTTCA	Amplification of *ramo5*
Mche_StrR_F Mche_StrR_R	TTTGATATCGGAGGGATCGAATGAAGGCGGAGC TTTGAATTCTGTCCGGCTCAGGCGCTGC	Amplification of *orf28*
pAm_seq_F pAm_seq_R	GATGTCATCAGCGGTGGAG TGAGCGGATAACAATTTCA	Verification of genes cloned into pSET152A
pTES_ver_F pTES_ver_R	CGCGTGTTCGTCGGGCTCTT GACCGAGCGCAGCGAGTCAG	Verification of genes cloned into pTES

## Data Availability

All strains and plasmids generated in this study are available from the corresponding author upon reasonable request.

## Supplementary Materials

The following supporting information can be downloaded at https://www.mdpi.com/article/10.3390/antibiotics13020115/s1. Figure S1: Multiple amino acid sequence alignment of 16 StrR-like PSRs that represent main clades of the phylogenetic tree in Figure 1 and structural models for NbrR10, FocG, QER91000, and DtpR2; Figure S2: Multiple amino acid sequence alignment of 13 StrR-like PSRs that represent main clades of the phylogenetic tree in Figure 1, where the secondary structures of the proteins are shown; Table S1: Information on StrR-like PSRs coded within LDP BGCs and BGCs available in MIBiG; Table S2: Summary of the StrR-like pathway-specific regulators of some GPA BGCs absent in MIBiG. References [2,14,19,29,37,41,42,50,53,55,66,68,72,73,74,75,76,77,78,79,80,81,82,83,84,85,86,87,88,89,90,91,92,93,94,95,96,97,98,99,100,101,102,103,104,105,106,107,108,109,110,111,112,113,114,115,116,117,118,119,120,121,122] are cited in the Supplementary Materials.
