# Supplementary material for: The Impact of Heterologous Regulatory Genes from Lipodepsipeptide Biosynthetic Gene Clusters on the Production of Teicoplanin and A40926
Source: Antibiotics (Basel). 2024 Jan 24;13(2):115. doi: 10.3390/antibiotics13020115 (PMC10886168; doi:10.3390/antibiotics13020115)
Supplement: Supplementary file 1 [file antibiotics-13-00115-s001.zip › antibiotics-2819291-supplementary.pdf]

Electronic Supplementary Materials

The impact of heterologous regulatory genes from lipodepsipeptide biosynthetic gene clusters on the production of teicoplanin and A40926

Kseniia Zhukrovska [1], Elisa Binda [2], Victor Fedorenko [1], Flavia Marinelli [2,*], and Oleksandr Yushchuk [1,2]

[1] Department of Genetics and Biotechnology, Ivan Franko National University of Lviv, Lviv, Ukraine

[2] Department of Biotechnology and Life Sciences, University of Insubria, Varese, Italy

* Correspondence: flavia.marinelli@uninsubria.it; Tel.: +39-0332-42-1546

Table S1. Information of StrR-like PSRs coded within LDP BGCs (A), as well as in antibiotic BGCs available in MIBiG (B).

MIBiG/GenBank accession number for the hit (1)	Protein ID (2)	E-value (3)	Amino acid sequence identity (4)	Microorganism (5)	Antibiotic (6)	Class (7)	Reference (8)
	Ramo5			*Actinoplanes ramoplaninifer* ATCC 33076	Ramoplanin	LDP	[1]
**(A) StrR-like pathway specific regulators from LDP BCGs**							
BGC0000341	End22 ABD65942	4.53e-58	49.2%	*Streptomyces fungicidicus* ATCC 21013	Enduracidin	LDP	[2]
BGC0000341	End24 ABD65944	2.28e-71	43.2%	*Streptomyces fungicidicus* ATCC 21013	Enduracidin	LDP	[2]
NZ_FMIB01000002	Chers28 WP_091305478	8.99e-128	71.6%	*Micromonospora chersina* DSM 44151	Chersina-mycin	LDP	[3]
NZ_KB913037	Not annotated	2.25e-71	47.2%	*Amycolatopsis balhimycina* FH 1894	Putative LDP	LDP	[3]
NZ_KB913037	Not annotated	9.46e-69	45.8%	*Amycolatopsis balhimycina* FH 1894	Putative LDP	LDP	[3]
CP016174	ANN21820	1.97e-71	45.8%	*Amycolatopsis orientalis* B-37	Putative LDP	LDP	[3]
CP016174	ANN21819	5.83e-69	45.2%	*Amycolatopsis orientalis* B-37	Putative LDP	LDP	[3]
NZ ASJB01000042	WP_235783490	4.77e-72	46.0%	*Amycolatopsis orientalis* DSM 40040/KCTC 9412	Putative LDP	LDP	[3]
NZ ASJB01000042	WP_235783491	1.02e-69	45.8%	*Amycolatopsis orientalis* DSM 40040/KCTC 9412	Putative LDP	LDP	[3]
LT629775	SDT43639.1	3.26e-56	41.1%	*Streptomyces* sp. TLI_053	Putative LDP	LDP	[3]
VFOE01000001	TQL19429	1.78e-72	49.2%	*Streptomyces sp.* SLBN-134 Ga0314649_11	Putative LDP	LDP	[3]
VFOE01000001	TQL19431	3.02e-63	43.1%	*Streptomyces sp.* SLBN-134 Ga0314649_11	Putative LDP	LDP	[3]
**(B) StrR-like pathway specific regulators of BCGs from MIBiG database**							
BGC0000021	ApoR3 AEP40926	1.53e-70	48.2%	*Nocardiopsis* sp. FU 40	Apoptolidin	Type I polyketide	[4]
BGC0001381	NbrR10 AJO72769	7.30e-49	39.3%	*Nocardia terpenica* IFM 0406	Brasilinolide	Type I polyketide	[5]
BGC0000159	TtmQ ABW96549	4.85e-53	41.3%	*Streptomyces spiroverticillatus* CGMCC:4.1749	Tautomycin	Modular type I polyketide	[6]

(1)	(2)	(3)	(4)	(5)	(6)	(7)	(8)
BGC0000112	AAM78007	2.14e-58	40.2%	*Streptomyces carzinostaticus* ssp. *neocarzinostaticus* ATCC 15944	Neocarzinostatin	Enediyne, type I polyketide	[7]
BGC0000965	AAL06695	2.44e-65	42.2%	*Streptomyces globisporus* C-1027	C-1027	Enediyne, type I polyketide	[8]
BGC0001008	MdpR1 ABY65997	1.20e-64	46.6%	*Actinomadura madurae* ATCC 39144	Maduropeptin	Enediyne, type I polyketide	[9]
BGC0001397	ALU98457	2.44e-65	42.2%	*Streptomyces globisporus* C-1027	C-1027	Enediyne, type I polyketide	[10]
BGC0001584	ANY94466	1.79e-64	42.5%	*Streptomyces* sp. CB02366	C-1027	Enediyne, type I polyketide	[11]
BGC0001904	ALJ99870	6.16e-67	44.2%	*Micromonospora rosaria* SCSIO N160	Fluostatin	Type II polyketide	[12]
BGC0000240	Lom15 AHZ61849	6.50e-53	37.8%	*Salinispora pacifica* DPJ-0016	Lomaiviticin	Type II polyketide	[13]
BGC0000241	ABP54638	9.22e-54	36.8%	*Salinispora tropica* CNB-440	Lomaiviticin	Type II polyketide	[13]
BGC0000267	SaqI ACP19349	1.68e-76	49.7%	*Micromonospora* sp. Tü 6368	Saquayamycin	Type II polyketide	[14]
BGC0001596	WP_15939 3028	9.99e-61	45.1%	*Streptomyces albus* DSM 41398	Fluostatins	Type II polyketide	[15]
BGC0001693	ARD70863	2.18e-58	40.9%	*Micromonospora echinospora* SCSIO 04089	Nenestatin	Type II polyketide	[16]
BGC0000690	StrR CAA07385	1.11e-72	46.5%	*Streptomyces glaucescens* GLA.0	Hydroxystreptomycin	Aminoglycoside	[17]
BGC0000698	ABC42540	9.85e-58	42.8%	*Streptomyces hygroscopicus* NRRL 2388	Hygromycin	Aminoglycoside	[18]
BGC0000702	BAD20753	1.18e-63	44.9%	*Streptomyces kanamyceticus* ATCC 12853	Kanamycin	Aminoglycoside	[19]
BGC0000716	SpcR AAB66654	2.15e-66	43.6%	*Streptomyces netropsis* JCM 4063	Spectinomycin	Aminoglycoside	[20]
BGC0000724	StrR BAG22760	3.37e-70	47.2%	*Streptomyces griseus* ssp. *griseus* NBRC 13350	Streptomycin	Aminoglycoside	[21]
BGC0000832	CloG AAN65222	1.92e-59	43.9%	*Streptomyces roseochromogenus subsp. oscitans* DS 12.976	Clorobiocin	Aminocoumarine	[22]
BGC0000833	CouG AAG29778	2.35e-56	41.3%	*Streptomyces rishiriensis* DSM 40489	Coumermycin	Aminocoumarine	[23]
BGC0000834	NovG AAF67500	4.28e-53	41.8%	*Streptomyces niveus* NCIMB 9219	Novobiocin	Aminocoumarine	[24]

(1)	(2)	(3)	(4)	(5)	(6)	(7)	(8)
BGC0001783	StnT2 AFW04588	2.79e-61	43.4%	*Streptomyces flocculus* CGMCC 4.1223	Streptonigrin	Aminoquinone alkaloid	[25]
BGC0000935	EsmT1 AFB35624	3.61e-62	43.4%	*Streptomyces antibioticus* Tü 2706	Esmeraldin	Phenazine	[26]
BGC0000679	EWM63064	1.08e-70	48.1%	*Micromonospora* sp. M42	Diazepinomicin	Terpene	[27]
BGC0002591	ARM20266	1.80e-59	46.5%	*Streptomyces* sp. NA04227	Aurachin	ladderane,terpene	[28]
BGC0001387	AMP46602	2.21e-60	41.7%	*Streptomyces calvus* ATCC 13382	Nucleocidin	Fluorinated ribonucleoside	[29]
BGC0001985	QER91000	6.25e-46	36.5%	*Streptomyces candidus* NRRL 3601	Pyrazofurin	C-nucleoside	[30]
BGC0002039	FocG AVW82900	1.99e-48	37.7%	*Nocardia interforma* ATCC 21072	Coformycin, formycin	C-nucleoside	[31]
BGC0001193	DtpR2 AJI44174	9.70e-76	48.6%	*Saccharothrix algeriensis* NRRL B-24137	Thiolutin	Dithiolopyrrolone	[32]
BGC0000893	CCA54200	7.10e-58	43.5%	*Streptomyces venezuelae* ATCC 10712	Chloramphenicol	NRP	[33]
BGC0001620	ASX95224	6.85e-70	47.9%	*Streptomyces atratus* SCSIO_ZH16	Ilamycin	Cyclic NRP	[34]
BGC0001763	RufA BBA20948	5.12e-70	47.9%	*Streptomyces atratus* SCSIO_ZH16	Rufomycin	Cyclic NRP	[35]
BGC0001233	FegB ALK27899	1.03e-66	43.5%	*Streptomyces* sp. DSM 11171	Feglymycin	Linear NRP	[36]
BGC0000311	Bbr CAG25754	6.57e-71	45.9%	*Amycolatopsis balhimycina* DSM 5908	Balhimycin	Type I GPA	[37]
BGC0000455	VtrR AEI58862	5.70e-74	47.2%	*Amycolatopsis orientalis* HCCB10007	Vancomycin	Type I GPA	[38]
BGC0001459	OKA09420	3.95e-71	45.6%	*Amycolatopsis regifaucium* GY080	Decaplanin	Type I GPA	[39]
BGC0001460	EME52993	1.28e-71	45.9%	*Amycolatopsis decaplanina* DSM 44594	Decaplanin	Type I GPA	[40]
BGC0001461	OLZ50883	5.07e-70	46.6%	*Amycolatopsis keratiniphila* ssp. *nogabecina* FH 1893	Nogabecin	Type II GPA	[41]
BGC0001462	OLZ52648	5.65e-71	47.0%	*Amycolatopsis coloradensis* DSM 44225	Avoparcin	Type II GPA	[42]
BGC0001955	AYA22339	4.13e-70	46.6%	*Amycolatopsis keratiniphila* NRRL B24117	Keratinimicin	Type II GPA	[43]

(1)	(2)	(3)	(4)	(5)	(6)	(7)	(8)
BGC0001178	AGS77330	8.30e-69	46.3%	*Actinoplanes* sp. ATCC 53533	UK-68,597	Type III GPA	[44]
BGC0000290	StaQ AAM80553	6.10e-67	43.7%	*Streptomyces toyocaensis* NRRL 15009	A47934	Type III GPA	[45]
BGC0000418	AIE77054	9.25e-73	47.2%	*Amycolatopsis lurida* NRRL 2430	Ristocetin	Type III GPA	[46]
BGC0000419	AIG79246	4.05e-72	46.9%	*Amycolatopsis japonica* MG417-CF17	Ristocetin	Type III GPA	[47]
BGC0000440	Tei15* CAE53369	5.08e-68	44.5%	*Actinoplanes teichomyceticus* ATCC 31121	Teicoplanin	Type IV GPA	[48]
BGC0002344	NocRII QYC40303	1.87e-72	45.9%	*Nonomuraea coxensis* DSM 45129	A50926	Type IV GPA	[49]
BGC0000289	Dbv4 CAD91199	2.35e-73	46.5%	*Nonomuraea gerenzanensis* ATCC 39727	A40926	Type IV GPA	[50]
BGC0002637	RSO11558	2.69e-78	48.4%	*Streptomyces* sp. WAC 06783	Rimomycin	Type V GPA	[51]
BGC0002638	MCC5036781	2.31e-78	47.7%	*Streptomyces* sp. WAC 00631	Misaugamycin	Type V GPA	[51]
BGC0001635	AQZ71349	1.28e-64	47.1%	*Nonomuraea* sp. ATCC 55076	Kistamicin	Type V GPA	[52]
BGC0000326	AAK81822	3.76e-68	45.5%	*Streptomyces lavendulae* SANK 60477	Complestatin	Type V GPA	[53]

Table S2. Summary of the StrR-like pathway specific regulators of some GPA BGCs absent in MIBiG.

GenBank accession number for the hit	Protein ID	E value	AA sequence identity	Organism	Antibiotic	Class	Reference
HM486074	ADU56075	2.59e-92	57.3%	Uncultured organism CA37	Putative GPA (predicted *in silico*)	GPA	[54]
HM486074	ADU56074	2.25e-89	54.5%	Uncultured organism CA37	Putative GPA (predicted *in silico*)	GPA	[54]
HM486074	ADU56064	3.41e-66	45.8%	Uncultured organism CA37	Putative GPA (predicted *in silico*)	GPA	[54]
HM486075	ADU56085	1.38e-72	45.9%	Uncultured organism CA878	Putative GPA (predicted *in silico*)	GPA	[54]
HM486076	ADU56159	4.61e-70	45.4%	Uncultured organism CA915	Putative GPA (predicted *in silico*)	GPA	[54]
KF264554	AGS49770	2.70e-67	45.2%	Uncultured bacterium esnapd15	Putative GPA (predicted *in silico*)	GPA	[55]
EU874253	ACJ60984	6.16e-74	46.5%	Uncultured soil bacterium clone D30	Putative GPA (predicted *in silico*)	GPA	[56]
EU874252	ACJ60943	8.97e-73	47.1%	Uncultured soil bacterium clone B128	Putative GPA (predicted *in silico*)	GPA	[56]
JX026280	AGF91741	9.20e-73	46.8%	*Streptomyces* sp. WAC1420	Pekiskomycin	Type I GPA	[57]
CP016174	ANN20081	3.93e-72	45.9%	*Amycolatopsis orientalis* B-37	Norvancomycin	Type I GPA	[58]
QHJI01000006	RSN30133	6.92e-75	47.2%	*Amycolatopsis* sp. WAC 04169	Dimethylvancomycin	Type I GPA	[59]
QHKI01000005	RSM88020	8.46e-74	47.9%	*Kibdelosporangium aridum* A82846	Chloroeremomycin	Type I GPA	[59]
JPLW01000007	KFZ77397	7.28e-73	46.9%	*Amycolatopsis* sp. MJM2582	Ristocetin	Type III GPA	[46]
CP024972	QXV59348	4.49e-69	46.5%	*Amycolatopsis sp.* TNS106	Ristocetin	Type III GPA	[60]
MUXN01000025	OOC02329	1.48e-72	46.5%	*Amycolatopsis azurea* DSM 43854	Azureomycin	Type III GPA	[61]
QHHX01000008	RSN28334	3.13e-73	47.2%	*Amycolatopsis* sp. WAC 01416	GP01416	Type IV GPA	[59]
CP029618	AZM51136	2.56e-75	49.2%	*Streptomyces* sp. WAC 06738	GP6738	Type IV GPA	[59]
JMGY01000008	KEF19256	2.32e-78	48.4%	*Streptomyces rimosus* R6-500MV9-R8	Rimomycin	Type V GPA	[51]
MCNU01000022	OFA57931	2.10e-74	46.4%	*Streptomyces fradiae* Olg4R	Miasugamycin	Type V GPA	[51]

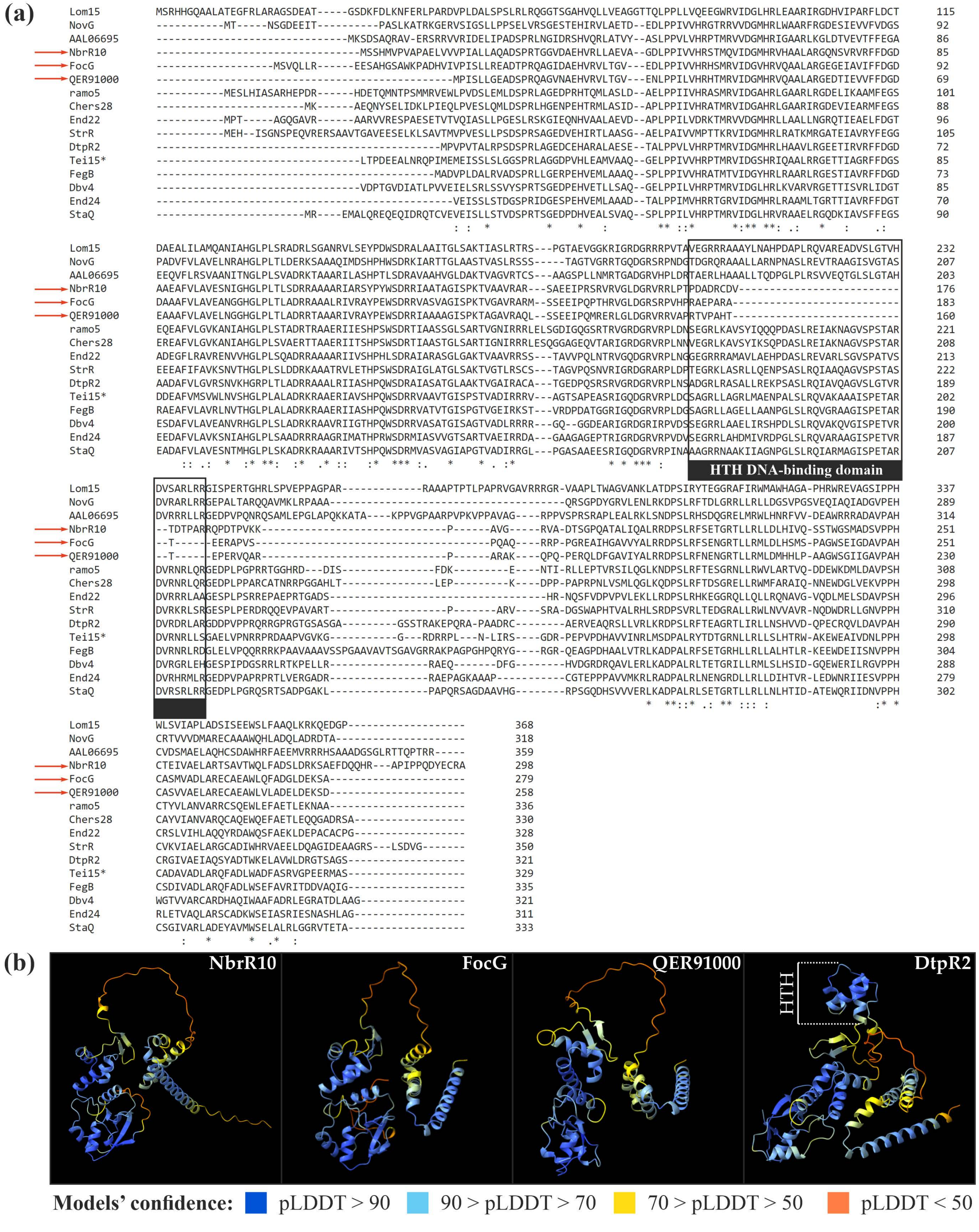

Figure S1. Multiple amino acid sequence alignment of 16 StrR-like PSRs that represent main clades of the phylogenetic tree from Figure S1 (a), showing that proteins of "C-nucleoside" clade – NbrR10, FocG, and QER91000 (marked with red arrows) – lack HTH DNA-binding domain, that is also demonstrated by 3D structure modelling (b). StrR-like PSR, phylogenetically closest to "C-nucleoside" clade (see Figure S1) – DtpR2 – retains HTH DNA-binding domain. Secondary and tertiary structure modelling was performed and visualized in CHIMERA X using AlphaFold [63–65]. Multiple amino acid sequence alignment was done using Clustal Omega [66].

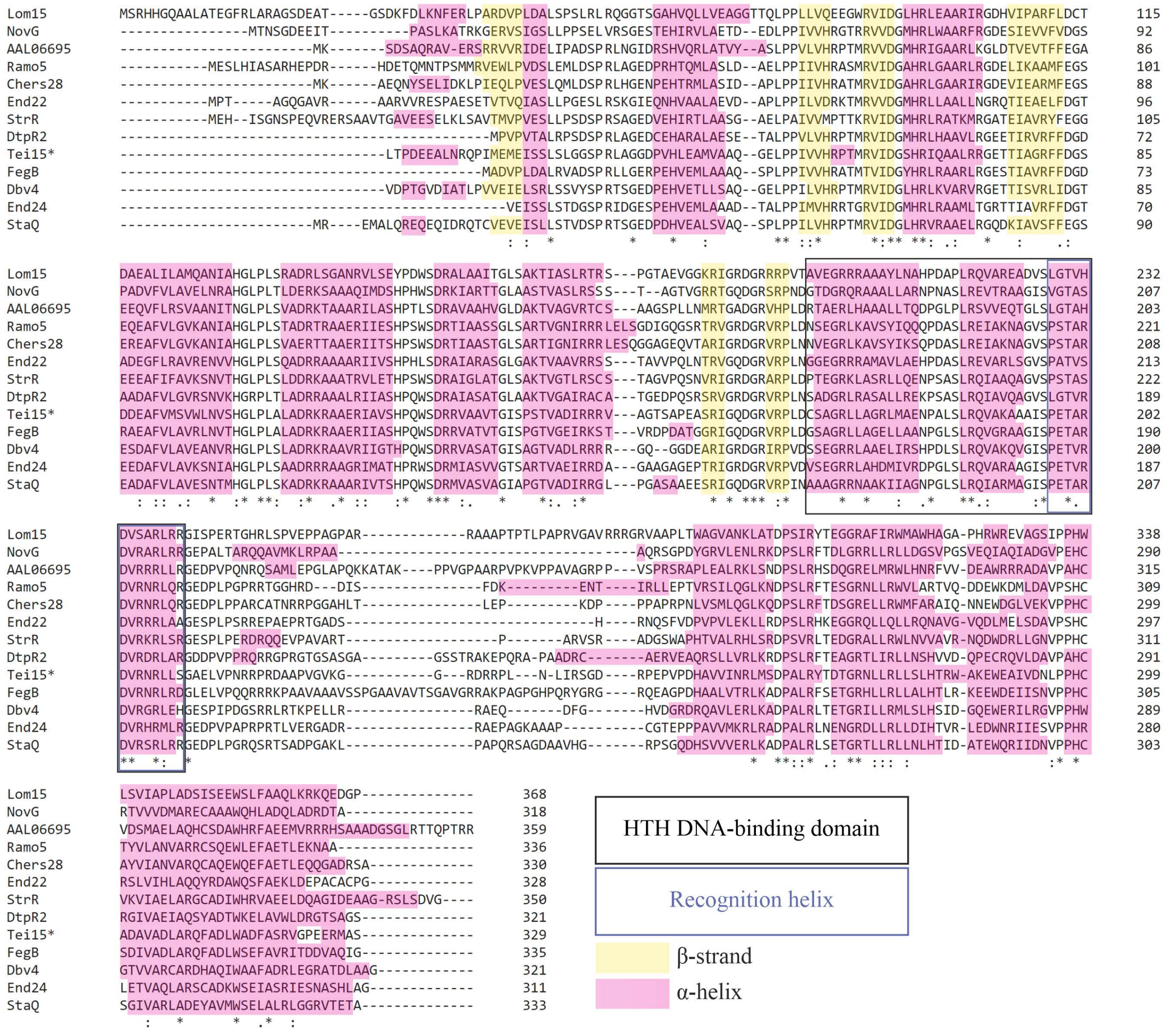

Figure S2. Multiple amino acid sequence alignment of 13 StrR-like PSRs that represent main clades of the phylogenetic tree from Figure S1, where the secondary structure of the proteins is shown. Secondary structure modelling was performed in CHIMERA X using AlphaFold [63–65]. Multiple amino acid sequence alignment was done using Clustal Omega [66].

Supplementary References

1. Farnet, C.M. GENE CLUSTER FOR RAMOPLANIN BIOSYNTHESIS 2005, 222.
2. Yin, X.; Zabriskie, T.M. The enduracidin biosynthetic gene cluster from Streptomyces fungicidicus. *Microbiology* **2006**, *152*, 2969–2983, doi:10.1099/mic.0.29043-0.
3. Morgan, K.T.; Zheng, J.; McCafferty, D.G. Discovery of Six Ramoplanin Family Gene Clusters and the Lipoglycodepsipeptide Chersinamycin**. *ChemBioChem* **2021**, *22*, 176–185, doi:10.1002/cbic.202000555.
4. Du, Y.; Derewacz, D.K.; Deguire, S.M.; Teske, J.; Ravel, J.; Sulikowski, G.A.; Bachmann, B.O. Biosynthesis of the apoptolidins in Nocardiopsis sp. FU 40. *Tetrahedron* **2011**, *67*, 6568–6575, doi:10.1016/J.TET.2011.05.106.
5. Chiu, H.T.; Weng, C.P.; Lin, Y.C.; Chen, K.H. Target-specific identification and characterization of the putative gene cluster for brasilinolide biosynthesis revealing the mechanistic insights and combinatorial synthetic utility of 2-deoxy-l-fucose biosynthetic enzymes. *Org. Biomol. Chem.* **2016**, *14*, 1988–2006, doi:10.1039/C5OB02292D.
6. Li, W.; Ju, J.; Rajski, S.R.; Osada, H.; Shen, B. Characterization of the tautomycin biosynthetic gene cluster from Streptomyces spiroverticillatus unveiling new insights into dialkylmaleic anhydride and polyketide biosynthesis. *J. Biol. Chem.* **2008**, *283*, 28607–28617, doi:10.1074/JBC.M804279200.
7. Liu, W.; Nonaka, K.; Nie, L.; Zhang, J.; Christenson, S.D.; Bae, J.; Van Lanen, S.G.; Zazopoulos, E.; Farnet, C.M.; Yang, C.F.; et al. The neocarzinostatin biosynthetic gene cluster from Streptomyces carzinostaticus ATCC 15944 involving two iterative type I polyketide synthases. *Chem. Biol.* **2005**, *12*, 293–302, doi:10.1016/J.CHEMBIOL.2004.12.013.
8. Liu, W.; Christenson, S.D.; Standage, S.; Shen, B. Biosynthesis of the enediyne antitumor antibiotic C-1027. *Science (80-.).* **2002**, *297*, 1170–1173, doi:10.1126/science.1072110.
9. Van Lanen, S.G.; Oh, T.J.; Liu, W.; Wendt-Pienkowski, E.; Shen, B. Characterization of the maduropeptin biosynthetic gene cluster from Actinomadura madurae ATCC 39144 supporting a unifying paradigm for enediyne biosynthesis. *J. Am. Chem. Soc.* **2007**, *129*, 13082–13094, doi:10.1021/JA073275O.
10. Li, X.; Lei, X.; Zhang, C.; Jiang, Z.; Shi, Y.; Wang, S.; Wang, L.; Hong, B. Complete genome sequence of Streptomyces globisporus C-1027, the producer of an enediyne antibiotic lidamycin. *J. Biotechnol.* **2016**, *222*, 9–10, doi:10.1016/j.jbiotec.2016.02.004.
11. Yan, X.; Ge, H.; Huang, T.; Hindra; Yang, D.; Teng, Q.; Crnovčić, I.; Li, X.; Rudolf, J.D.; Lohman, J.R.; et al. Strain prioritization and genome mining for enediyne natural products. *MBio* **2016**, *7*, doi:10.1128/mBio.02104-16.

12. Yang, C.; Huang, C.; Zhang, W.; Zhu, Y.; Zhang, C. Heterologous Expression of Fluostatin Gene Cluster Leads to a Bioactive Heterodimer. *Org. Lett.* **2015**, *17*, 5324–5327, doi:10.1021/ACS.ORGLETT.5B02683.

13. Janso, J.E.; Haltli, B.A.; Eustáquio, A.S.; Kulowski, K.; Waldman, A.J.; Zha, L.; Nakamura, H.; Bernan, V.S.; He, H.; Carter, G.T.; et al. Discovery of the lomaiviticin biosynthetic gene cluster in Salinispora pacifica. *Tetrahedron* **2014**, *70*, 4156–4164, doi:10.1016/j.tet.2014.03.009.

14. Erb, A.; Luzhetskyy, A.; Hardter, U.; Bechthold, A. Cloning and sequencing of the biosynthetic gene cluster for saquayamycin Z and galtamycin B and the elucidation of the assembly of their saccharide chains. *Chembiochem* **2009**, *10*, 1392–1401, doi:10.1002/CBIC.200900054.

15. Jin, J.; Yang, X.; Liu, T.; Xiao, H.; Wang, G.; Zhou, M.; Liu, F.; Zhang, Y.; Liu, D.; Chen, M.; et al. Fluostatins M-Q Featuring a 6-5-6-6 Ring Skeleton and High Oxidized A-Rings from Marine Streptomyces sp. PKU-MA00045. *Mar. Drugs* **2018**, *16*, doi:10.3390/MD16030087.

16. Jiang, X.; Zhang, Q.; Zhu, Y.; Nie, F.; Wu, Z.; Yang, C.; Zhang, L.; Tian, X.; Zhang, C. Isolation, structure elucidation and biosynthesis of benzo[b]fluorene nenestatin A from deep-sea derived Micromonospora echinospora SCSIO 04089. *Tetrahedron* **2017**, *73*, 3585–3590, doi:10.1016/J.TET.2017.03.054.

17. Beyer, S.; Distler, J.; Piepersberg, W. The str gene cluster for the biosynthesis of 5′-hydroxystreptomycin in Streptomyces glaucescens GLA.0 (ETH 22794): New operons and evidence for pathway-specific regulation by StrR. *Mol. Gen. Genet.* **1996**, *250*, 775–784, doi:10.1007/BF02172990.

18. Palaniappan, N.; Ayers, S.; Gupta, S.; Habib, E.S.; Reynolds, K.A. Production of hygromycin A analogs in Streptomyces hygroscopicus NRRL 2388 through identification and manipulation of the biosynthetic gene cluster. *Chem. Biol.* **2006**, *13*, 753–764, doi:10.1016/J.CHEMBIOL.2006.05.013.

19. Yanai, K.; Murakami, T. The kanamycin biosynthetic gene cluster from Streptomyces kanamyceticus. *J. Antibiot. (Tokyo).* **2004**, *57*, 351–354, doi:10.7164/ANTIBIOTICS.57.351.

20. Lyutzkanova, D.; Distler, J.; Altenbuchner, J. A spectinomycin resistance determinant from the spectinomycin producer Streptomyces flavopersicus. *Microbiology* **1997**, *143 (Pt 7*, 2135–2143, doi:10.1099/00221287-143-7-2135.

21. Ohnishi, Y.; Ishikawa, J.; Hara, H.; Suzuki, H.; Ikenoya, M.; Ikeda, H.; Yamashita, A.; Hattori, M.; Horinouchi, S. Genome sequence of the streptomycin-producing microorganism Streptomyces griseus IFO 13350. *J. Bacteriol.* **2008**, *190*, 4050–4060, doi:10.1128/JB.00204-08.

22. Pojer, F.; Li, S.M.; Heide, L. Molecular cloning and sequence analysis of the clorobiocin biosynthetic gene cluster: New insights into the biosynthesis of aminocoumarin

antibiotics. *Microbiology* **2002**, *148*, 3901–3911, doi:10.1099/00221287-148-12-3901.

23. Wang, Z.X.; Li, S.M.; Heide, L. Identification of the coumermycin A1 biosynthetic gene cluster of streptomyces rishiriensis DSM 40489. *Antimicrob. Agents Chemother.* **2000**, *44*, 3040–3048, doi:10.1128/AAC.44.11.3040-3048.2000.

24. Steffensky, M.; Mühlenweg, A.; Wang, Z.X.; Li, S.M.; Heide, L. Identification of the novobiocin biosynthetic gene cluster of Streptomyces spheroides NCIB 11891. *Antimicrob. Agents Chemother.* **2000**, *44*, 1214–1222, doi:10.1128/AAC.44.5.1214-1222.2000.

25. Xu, F.; Kong, D.; He, X.; Zhang, Z.; Han, M.; Xie, X.; Wang, P.; Cheng, H.; Tao, M.; Zhang, L.; et al. Characterization of streptonigrin biosynthesis reveals a cryptic carboxyl methylation and an unusual oxidative cleavage of a N-C bond. *J. Am. Chem. Soc.* **2013**, *135*, 1739–1748, doi:10.1021/JA3069243.

26. Rui, Z.; Ye, M.; Wang, S.; Fujikawa, K.; Akerele, B.; Aung, M.; Floss, H.G.; Zhang, W.; Yu, T.W. Insights into a divergent phenazine biosynthetic pathway governed by a plasmid-born esmeraldin gene cluster. *Chem. Biol.* **2012**, *19*, 1116–1125, doi:10.1016/J.CHEMBIOL.2012.07.025.

27. McAlpine, J.B.; Banskota, A.H.; Charan, R.D.; Schlingmann, G.; Zazopoulos, E.; Piraee, M.; Janso, J.; Bernan, V.S.; Aouidate, M.; Farnet, C.M.; et al. Biosynthesis of diazepinomicin/ECO-4601, a Micromonospora secondary metabolite with a novel ring system. *J. Nat. Prod.* **2008**, *71*, 1585–1590, doi:10.1021/NP800376N.

28. Zhang, M.; Yang, C.L.; Xiao, Y.S.; Zhang, B.; Deng, X.Z.; Yang, L.; Shi, J.; Wang, Y.S.; Li, W.; Jiao, R.H.; et al. Aurachin SS, a new antibiotic from Streptomyces sp. NA04227. *J. Antibiot. (Tokyo).* **2017**, *70*, 853–855, doi:10.1038/JA.2017.50.

29. Zhu, X.M.; Hackl, S.; Thaker, M.N.; Kalan, L.; Weber, C.; Urgast, D.S.; Krupp, E.M.; Brewer, A.; Vanner, S.; Szawiola, A.; et al. Biosynthesis of the Fluorinated Natural Product Nucleocidin in Streptomyces calvus Is Dependent on the bldA-Specified Leu-tRNA(UUA) Molecule. *Chembiochem* **2015**, *16*, 2498–2506, doi:10.1002/CBIC.201500402.

30. Zhao, G.; Yao, S.; Rothchild, K.W.; Liu, T.; Liu, Y.; Lian, J.; He, H.Y.; Ryan, K.S.; Du, Y.L. The Biosynthetic Gene Cluster of Pyrazomycin—A C-Nucleoside Antibiotic with a Rare Pyrazole Moiety. *ChemBioChem* **2020**, *21*, 644–649, doi:10.1002/CBIC.201900449.

31. Zhang, M.; Zhang, P.; Xu, G.; Zhou, W.; Gao, Y.; Gong, R.; Cai, Y.S.; Cong, H.; Deng, Z.; Price, N.P.J.; et al. Comparative Investigation into Formycin A and Pyrazofurin A Biosynthesis Reveals Branch Pathways for the Construction of C-Nucleoside Scaffolds. *Appl. Environ. Microbiol.* **2020**, *86*, doi:10.1128/AEM.01971-19.

32. Huang, S.; Tong, M.; Qin, Z.; Deng, Z.; Deng, H.; Yu, Y. Identification and characterization of the biosynthetic gene cluster of thiolutin, a tumor angiogenesis inhibitor, in Saccharothrix algeriensis NRRL B-24137. *Anticancer. Agents Med. Chem.* **2015**, *15*, 277–284, doi:10.2174/1871520614666141027145200.

33. He, J.; Magarvey, N.; Piraee, M.; Vining, L.C. The gene cluster for chloramphenicol biosynthesis in Streptomyces venezuelae ISP5230 includes novel shikimate pathway homologues and a monomodular non-ribosomal peptide synthetase gene. *Microbiology* **2001**, *147*, 2817–2829, doi:10.1099/00221287-147-10-2817.

34. Ma, J.; Huang, H.; Xie, Y.; Liu, Z.; Zhao, J.; Zhang, C.; Jia, Y.; Zhang, Y.; Zhang, H.; Zhang, T.; et al. Biosynthesis of ilamycins featuring unusual building blocks and engineered production of enhanced anti-tuberculosis agents. *Nat. Commun.* **2017**, *8*, doi:10.1038/S41467-017-00419-5.

35. Tomita, H.; Katsuyama, Y.; Minami, H.; Ohnishi, Y. Identification and characterization of a bacterial cytochrome P450 monooxygenase catalyzing the 3-nitration of tyrosine in rufomycin biosynthesis. *J. Biol. Chem.* **2017**, *292*, 15859–15869, doi:10.1074/JBC.M117.791269.

36. Gonsior, M.; Mühlenweg, A.; Tietzmann, M.; Rausch, S.; Poch, A.; Süssmuth, R.D. Biosynthesis of the peptide antibiotic feglymycin by a linear nonribosomal peptide synthetase mechanism. *ChemBioChem* **2015**, *16*, 2610–2614, doi:10.1002/cbic.201500432.

37. Pelzer, S.; Süßmuth, R.; Heckmann, D.; Recktenwald, J.; Huber, P.; Jung, G.; Wohlleben, W. Identification and analysis of the balhimycin biosynthetic gene cluster and its use for manipulating glycopeptide biosynthesis in Amycolatopsis mediterranei DSM5908. *Antimicrob. Agents Chemother.* **1999**, *43*, 1565–1573, doi:10.1128/aac.43.7.1565.

38. Xu, L.; Huang, H.; Wei, W.; Zhong, Y.; Tang, B.; Yuan, H.; Zhu, L.; Huang, W.; Ge, M.; Yang, S.; et al. Complete genome sequence and comparative genomic analyses of the vancomycin-producing Amycolatopsis orientalis. *BMC Genomics* **2014**, *15*, 1–18, doi:10.1186/1471-2164-15-363.

39. Tan, G.Y.A.; Robinson, S.; Lacey, E.; Brown, R.; Kim, W.; Goodfellow, M. Amycolatopsis regifaucium sp. nov., a novel actinomycete that produces kigamicins. *Int. J. Syst. Evol. Microbiol.* **2007**, *57*, 2562–2567, doi:10.1099/IJS.0.64974-0.

40. Kaur, N.; Kumar, S.; Bala, M.; Raghava, G.P.S.; Mayilraj, S. Draft genome sequence of Amycolatopsis decaplanina strain DSM 44594T. *Genome Announc.* **2013**, *1*, e0013813, doi:10.1128/genomeA.00138-13.

41. Wink, J.M.; Kroppenstedt, R.M.; Ganguli, B.N.; Nadkarni, S.R.; Schumann, P.; Seibert, G.; Stackebrandt, E. Three new antibiotic producing species of the genus Amycolatopsis, Amycolatopsis balhimycina sp. nov., A. tolypomycina sp. nov., A. vancoresmycina sp. nov., and description of Amycolatopsis keratiniphila subsp. keratiniphila subsp. nov. and A. keratiniphi. *Syst. Appl. Microbiol.* **2003**, *26*, 38–46, doi:10.1078/072320203322337290.

42. Labeda, D.P. Amycolatopsis coloradensis sp. nov., the avoparcin (LL-AV290)-producing strain. *Int. J. Syst. Bacteriol.* **1995**, *45*, 124–127, doi:10.1099/00207713-45-1-124/CITE/REFWORKS.

43. Xu, F.; Wu, Y.; Zhang, C.; Davis, K.M.; Moon, K.; Bushin, L.B.; Seyedsayamdost, M.R.

A genetics-free method for high-throughput discovery of cryptic microbial metabolites. *Nat. Chem. Biol.* **2019**, *15*, 161–168, doi:10.1038/s41589-018-0193-2.

44. Yim, G.; Kalan, L.; Koteva, K.; Thaker, M.N.; Waglechner, N.; Tang, I.; Wright, G.D. Harnessing the synthetic capabilities of glycopeptide antibiotic tailoring enzymes: Characterization of the UK-68, 597 biosynthetic cluster. *ChemBioChem* **2014**, *15*, 2613–2623, doi:10.1002/cbic.201402179.

45. Pootoolal, J.; Thomas, M.G.; Marshall, C.G.; Neu, J.M.; Hubbard, B.K.; Walsh, C.T.; Wright, G.D. Assembling the glycopeptide antibiotic scaffold: The biosynthesis of A47934 from streptomyces toyocaensis NRRL15009. *Proc. Natl. Acad. Sci. U. S. A.* **2002**, *99*, 8962–8967, doi:10.1073/pnas.102285099.

46. Truman, A.W.; Kwun, M.J.; Cheng, J.; Yang, S.H.; Suh, J.W.; Hong, H.J. Antibiotic resistance mechanisms inform discovery: Identification and characterization of a novel amycolatopsis strain producing ristocetin. *Antimicrob. Agents Chemother.* **2014**, *58*, 5687–5695, doi:10.1128/AAC.03349-14.

47. Spohn, M.; Kirchner, N.; Kulik, A.; Jochim, A.; Wolf, F.; Muenzer, P.; Borst, O.; Gross, H.; Wohlleben, W.; Stegmann, E. Overproduction of ristomycin a by activation of a silent gene cluster in amycolatopsis japonicum mg417-cf17. *Antimicrob. Agents Chemother.* **2014**, *58*, 6185–6196, doi:10.1128/AAC.03512-14.

48. Sosio, M.; Kloosterman, H.; Bianchi, A.; de Vreugd, P.; Dijkhuizen, L.; Donadio, S. Organization of the teicoplanin gene cluster in Actinoplanes teichomyceticus. *Microbiology* **2004**, *150*, 95–102, doi:10.1099/mic.0.26507-0.

49. Yushchuk, O.; Vior, N.M.; Andreo-Vidal, A.; Berini, F.; Rückert, C.; Busche, T.; Binda, E.; Kalinowski, J.; Truman, A.W.; Marinelli, F. Genomic-Led Discovery of a Novel Glycopeptide Antibiotic by Nonomuraea coxensis DSM 45129. *ACS Chem. Biol.* **2021**, *16*, 915–928, doi:10.1021/acschembio.1c00170.

50. Sosio, M.; Stinchi, S.; Beltrametti, F.; Lazzarini, A.; Donadio, S. The gene cluster for the biosynthesis of the glycopeptide antibiotic A40926 by Nonomuraea species. *Chem. Biol.* **2003**, *10*, 541–549, doi:10.1016/S1074-5521(03)00120-0.

51. Xu, M.; Wang, W.; Waglechner, N.; Culp, E.J.; Guitor, A.K.; Wright, G.D. Phylogeny-Informed Synthetic Biology Reveals Unprecedented Structural Novelty in Type v Glycopeptide Antibiotics. *ACS Cent. Sci.* **2022**, *8*, 615–626, doi:10.1021/acscentsci.1c01389.

52. Nazari, B.; Forneris, C.C.; Gibson, M.I.; Moon, K.; Schramma, K.R.; Seyedsayamdost, M.R. Nonomuraea sp. ATCC 55076 harbours the largest actinomycete chromosome to date and the kistamicin biosynthetic gene cluster. *Medchemcomm* **2017**, *8*, 780–788, doi:10.1039/c6md00637j.

53. Chiu, H.T.; Hubbard, B.K.; Shah, A.N.; Eide, J.; Fredenburg, R.A.; Walsh, C.T.; Khosla, C. Molecular cloning and sequence analysis of the complestatin biosynthetic gene cluster. *Proc. Natl. Acad. Sci. U. S. A.* **2001**, *98*, 8548–8553, doi:10.1073/pnas.151246498.

54. Banik, J.J.; Craig, J.W.; Calle, P.Y.; Brady, S.F. Tailoring enzyme-rich environmental DNA clones: A source of enzymes for generating libraries of unnatural natural products. *J. Am. Chem. Soc.* **2010**, *132*, 15661–15670, doi:10.1021/ja105825a.

55. Owen, J.G.; Reddy, B.V.B.; Ternei, M.A.; Charlop-Powers, Z.; Calle, P.Y.; Kim, J.H.; Brady, S.F. Mapping gene clusters within arrayed metagenomic libraries to expand the structural diversity of biomedically relevant natural products. *Proc. Natl. Acad. Sci. U. S. A.* **2013**, *110*, 11797–11802, doi:10.1073/pnas.1222159110.

56. Banik, J.J.; Brady, S.F. Cloning and characterization of new glycopeptide gene clusters found in an environmental DNA megalibrary. *Proc. Natl. Acad. Sci. U. S. A.* **2008**, *105*, 17273–17277, doi:10.1073/pnas.0807564105.

57. Thaker, M.N.; Wang, W.; Spanogiannopoulos, P.; Waglechner, N.; King, A.M.; Medina, R.; Wright, G.D. Identifying producers of antibacterial compounds by screening for antibiotic resistance. *Nat. Biotechnol.* **2013**, *31*, 922–927, doi:10.1038/nbt.2685.

58. Lei, X.; Yuan, F.; Shi, Y.; Li, X.; Wang, L.; Hong, B. Draft genome sequence of norvancomycin-producing strain Amycolatopsis orientalis CPCC200066. *Genome Announc.* **2015**, *3*, doi:10.1128/genomeA.00296-15.

59. Waglechner, N.; McArthur, A.G.; Wright, G.D. Phylogenetic reconciliation reveals the natural history of glycopeptide antibiotic biosynthesis and resistance. *Nat. Microbiol.* **2019**, *4*, 1862–1871, doi:10.1038/s41564-019-0531-5.

60. Liu, K.; Hu, X.R.; Zhao, L.X.; Wang, Y.; Deng, Z.; Taoa, M. Enhancing Ristomycin A Production by Overexpression of ParBLike StrR Family Regulators Controlling the Biosynthesis Genes. *Appl. Environ. Microbiol.* **2021**, *87*, 1–19, doi:10.1128/AEM.01066-21.

61. Khatri, I.; Subramanian, S.; Mayilraj, S. Genome sequencing and annotation of Amycolatopsis azurea DSM 43854T. *Genomics Data* **2014**, *2*, 44–45, doi:10.1016/j.gdata.2013.12.003.

62. Tamura, K.; Stecher, G.; Kumar, S. MEGA11: Molecular Evolutionary Genetics Analysis Version 11. *Mol. Biol. Evol.* **2021**, *38*, 3022–3027, doi:10.1093/molbev/msab120.

63. Pettersen, E.F.; Goddard, T.D.; Huang, C.C.; Meng, E.C.; Couch, G.S.; Croll, T.I.; Morris, J.H.; Ferrin, T.E. UCSF ChimeraX: Structure visualization for researchers, educators, and developers. *Protein Sci.* **2021**, *30*, 70–82, doi:10.1002/pro.3943.

64. Mirdita, M.; Schütze, K.; Moriwaki, Y.; Heo, L.; Ovchinnikov, S.; Steinegger, M. ColabFold: making protein folding accessible to all. *Nat. Methods* **2022**, *19*, 679–682, doi:10.1038/s41592-022-01488-1.

65. Jumper, J.; Evans, R.; Pritzel, A.; Green, T.; Figurnov, M.; Ronneberger, O.; Tunyasuvunakool, K.; Bates, R.; Žídek, A.; Potapenko, A.; et al. Highly accurate protein structure prediction with AlphaFold. *Nature* **2021**, *596*, 583–589,

doi:10.1038/s41586-021-03819-2.

66. Sievers, F.; Higgins, D.G. Clustal Omega. *Curr. Protoc. Bioinforma.* **2014**, *2014*, 3.13.1-3.13.16, doi:10.1002/0471250953.bi0313s48.
